# Effect of *Uncaria tomentosa* extract on purinergic enzyme activities in lymphocytes of rats submitted to experimental adjuvant arthritis model

**DOI:** 10.1186/s12906-015-0694-4

**Published:** 2015-06-20

**Authors:** Lívia G. Castilhos, João F. P. Rezer, Jader B. Ruchel, Maria Luiza Thorstenberg, Jeandre A. dos S. Jaques, Josiane B. Schlemmer, Pedro H. Doleski, Mateus F. Rossato, Mariane A. da Silva, Emerson André Casalli, Ritiel Corrêa da Cruz, Juliano Ferreira, Margareth L. Athayde, Jamile F. Gonçalves, Daniela B. R. Leal

**Affiliations:** Departamento de Microbiologia e Parasitologia, Centro de Ciências da Saúde, Universidade Federal deSanta Maria, Av. Roraima, Prédio 20 – Sala 4102, 97105-900 Santa Maria, RS Brazil; Departamento de Química, Centro de Ciências Naturais e Exatas, Universidade Federal de Santa Maria, Av. Roraima, 97105-900 Santa Maria, RS Brazil; Departamento de Ciências Morfológicas, Instituto de Ciências Básicas da Saúde, UFRGS, Laboratório de Estudos Sobre as Alterações Celulares e Teciduais, Porto Alegre, RS Brazil; Laboratório de Enzimologia Aplicada ao Sistema Purinérgico, Departamento de Bioquímica, Instituto deCiências Básicas da Saúde, UFRGS, Porto Alegre, RS Brazil; Departamento de Farmácia Industrial, Universidade Federal de Santa Maria, Av. Roraima, 97105-900 Santa Maria, RS Brazil

**Keywords:** Adjuvant arthritis, *Uncaria tomentosa*, lymphocytes, E-NTPDase, Adenosine deaminase

## Abstract

**Background:**

Considering that adjuvant arthritis is an experimental model of arthritis widely used for preclinical testing of numerous anti-arthritic agents, which were taken by a large number of patients worldwide, it is of great interest to investigate the therapeutic action of compounds with anti-inflammatory properties, such as *Uncaria tomentosa* extract. Moreover, there are no studies demonstrating the effect of *U. tomentosa* on the metabolism of adenine nucleotides published so far. Thus, the purpose of the present study is to investigate the effects of *U. tomentosa* extract on E-NTPDase and E-ADA activities in lymphocytes of Complete Freund’s Adjuvant (CFA) arthritis induced rats.

**Methods:**

To evaluate the effect of *U. tomentosa* extract on the activity of E-NTPDase and ADA in lymphocytes, the rats were submitted to an experimental adjuvant arthritis model. Peripheral lymphocytes were isolated and E-NTPDase and E-ADA activities were determined. Data were analyzed by a one- or two-way ANOVA. Post hoc analyses were carried out by the Student-Newman-Keuls (SNK) Multiple Comparison Test.

**Results:**

E-NTPDase activity was increased in arthritic untreated. Arthritic rats which received *U. tomentosa* extract, presented similar results to the control group. However, results obtained for adenosine hydrolysis by E-ADA were not altered in arthritic rats. *U. tomentosa* extract did not alter E-NTPDase and E-ADA activity in healthy animals.

**Conclusions:**

The present investigation supports the hypothesis that the increased E-NTPDase activity verified in arthritic rats might be an attempt to maintain basal levels of ATP and ADP in the extracellular medium, since the arthritis induction causes tissue damage and, consequently, large amounts of ATP are released into this milieu. Also, it highlights the possibility to use *U. tomentosa* extract as an adjuvant to treat arthritis.

## Background

*Uncaria tomentosa* (Willd.) DC. is a giant vine of the Rubiaceae family that grows in the Amazon rainforest and because of its curved thorns it is commonly known as ‘cat’s claw’ or ‘uña de gato’. This species has been extensively used among several Peruvian tribes for the treatment of many diseases, such as arthritis and other inflammatory disorders [1]. Its active ingredients appear to be polyphenols (flavonoids, proanthocyanidins, and tannins), sterols, and alkaloids [2]. Among numerous *U. tomentosa* compounds, the mitraphylline is considered the major alkaloid present in this plant and it might be, at least partially, responsible for the anti-inflammatory activity of *Uncaria* bark extracts [3]. In most studies, its anti-inflammatory activity has been uniquely attributed to tetracyclic and pentacyclic oxindole alkaloids. However, currently available pharmacological data have indicated that this biological activity is due to the synergistic action of several compounds present in this species [4–8].

Arthritis is a systemic inflammatory disease characterized by joint pain, stiffness and swelling due to synovial inflammation, as well as fatigue and limitation in physical function, and increased morbidity and mortality [9]. It is a debilitating condition occurring at any age, peaking between the ages of 35 and 50 years and affecting around 1 % of the world population [10]. Adjuvant-induced arthritis is a commonly used model of inflammatory arthritis, which has an incidence of approximately 90 %, making it an ideal model to investigate arthritic changes and to evaluate compounds that may be useful for arthritis treatment [11, 12]. During the inflammatory process in arthritis, the immune and inflammatory responses are active, and it is well known that an imbalance between pro- and anti-inflammatory cytokine activities favors the induction of autoimmunity, chronic inflammation and thereby joint damage [13].

Extracellular nucleotides are essential molecules for the onset and maintenance of inflammatory reactions, whereas they are important signaling molecules [14]. The purinergic signaling system plays an important role in modulating the inflammatory and immune responses by extracellular biomolecules, such as adenine nucleotides (ATP, ADP and AMP) and their derived nucleoside adenosine [15]. There is evidence indicating that high extracellular ATP levels act through specific cell surface receptors as pro-inflammatory agents that potentiate the release of pro-inflammatory cytokines [16] from activated lymphocytes [17].

Extracellular ATP and adenosine levels, as well as the subsequent purinergic signaling, can be physiologically and dynamically controlled by the action of enzymes expressed in immune cells [16]. E-NTPDase (CD39) is the membrane-bound enzyme involved in the breakdown of ATP and ADP to AMP, which is sequentially hydrolyzed by 5′-nucleotidase to adenosine [18–20]. CD39 was first described as a B lymphocyte activation marker [21]. In leucocytes, its modulatory effects in cytokines expression, inflammatory response, cell-cell adhesion, as well as cell proliferation, via modulation of ATP levels in the pericellular milieu has been demonstrated [4–7]. According to Barankiewicz et al. [22], the presence of CD73 on the external surface of B cells is also related to B cell development.

E-ADA is another important enzyme that catalyzes the irreversible deamination of adenosine and 2′-deoxyadenosine to inosine and 2′-deoxyinosine, respectively. Therefore it contributes to the removal of adenosine from the extracellular compartment [23]. This enzyme has fundamental biological role in the proliferation and differentiation of lymphoid cells, particularly T lymphocytes, and maturation of monocytes [24], performing an important function in the immune system and inflammatory processes [25].

Considering that adjuvant arthritis is an experimental model of arthritis widely used for preclinical testing of numerous anti-arthritic agents, which are either under preclinical or clinical investigation, it is of clinical interest to investigate the therapeutic action of compounds with anti-inflammatory properties, such as *Uncaria tomentosa* extract. Moreover, to our knowledge, the current study is the first one evaluating the effect of *U. tomentosa* on the metabolism of adenine nucleotides. Thus, it is relevant to investigate its effects on the activity of E-NTPDase and E-ADA in lymphocytes of rats with Complete Freund’s Adjuvant (CFA)-induced arthritis.

## Methods

### Chemicals

Complete Freund’s Adjuvant (CFA - 0.6 % suspension of heat-killed *Mycobacterium tuberculosis* in liquid paraffin), 5-(N,N-diethylamino) pentyl-3,4,5-trimethoxybenzoate (TMB), hexadecyltrimethylammonium bromide (HTAB), the substrates ATP, ADP, adenosine, as well as Trizma base, Coomassie Brilliant Blue G and bovine serum albumin were obtained from Sigma Chemical Co (St. Louis, MO, USA) and K_2_HPO_4_, from Reagen. The acetonitrile and acetic acid were obtained from JTBarker, the tri-ethylamine and polyamide from Fluka and ethanol from Vetec. All the other chemicals used in this experiment were of the highest purity.

### Animals

Twenty eight adult female Wistar rats (200–300 g) were used in this experiment. Animals were divided into four groups, namely, control (C); extract (E); arthritis (A); and arthritis along with extract (A + E). Animals were kept on a 12-h light/12-h dark cycle, at a temperature of 22 ± 2 °C, with free access to food and water. The animals were handled according to the guidelines of the Committee on Brazilian Society of Animal Science Lab [26], in accordance with international guidelines. Furthermore, this investigation was approved by the Committee on the Use and Care of Laboratory Animals of our university (n. 125/2010(2)).

### Induction of arthritis

To investigate the effect of the *U. tomentosa* extract over the inflammatory process, the adjuvant-induced arthritis model was used and described by Sauzem et al., [27]. Animals were slightly anesthetized with isoflurane and 100 μL of Complete Freund Adjuvant (CFA - 0.6 % suspension of heat-killed *Mycobacterium tuberculosis* in liquid paraffin) was injected into the right hind paw to induce arthritis.

### HPLC analysis of *U. tomentosa* extract

Sample extraction was performed using a Unique® ultrasound, model USC 5000A, 40 kHz. Chromatographic analyses were performed on the Agilent 1100 HPLC system and a Zorbax® XDB C-18 column (150 mm × 4.6 mm, 3.5 _m Agilent) at 15 °C. Samples (80 mg) were diluted in 60 % ethanol (10 mL) and subjected to sonication (20 min at 30 °C). Following this step, 2 mL of sample were passed through a column containing 200 mg of polyamide, and the eluate was injected into an HPLC system. Separation was achieved using gradient elution of water (0.2 % acetic acid) adjusted to pH 6.9 with triethylamine (A) and acetonitrile (B) at a flow rate of 0.8 mL/min, detection was performed at 245 nm, and the concentrations of alkaloids were calculated as previously described [28].

### Treatment with *U. tomentosa* extract

The treatment of animals with extract began 15 days after induction of arthritis by CFA. The *U. tomentosa* root dry extract was donated by Herbarium Botanical Laboratory, PR-Brazil, lot number 991260. The organoleptic, physicochemical and microbiological characteristics were within quality standards; according to the Certificate of Analysis number 3301/11 presented by supplier. The extract was prepared daily with distilled water as vehicle and administered to the groups E and A + E by gavage twice a day at the dose of 150 mg/kg for 45 days, mimicking the Unha de Gato® phytotherapeutic from Herbarium Botanical Laboratory, indicated for treatment of patients with arthritis. Groups C and A received only distilled water in the same condition.

### Evidences of arthritis induction

Evidence of arthritis induction as mechanical sensitivity and paw thickness of each rat were evaluated briefly before induction of arthritis by CFA, and then 15 days after induction. Increased mechanical sensitivity and paw thickness were considered as markers of the inflammatory process. Moreover, these measurements were made 45 days after *U. tomentosa* treatment to observe the effect of *U. tomentosa* on the inflammatory process. To observe the development of edema, animals were held and the right hind paw thickness was measured using a digital caliper [29]. Additionally, increased paw thickness was considered as formation of edema. Mechanical allodynia was evaluated using the up-and-down method, described by Dixon [30], using von Frey filaments. Briefly, rats were placed in cages with a wire mesh bottom which allowed full access to the paws. The paw was touched with 1 of a series of 7 von Frey hairs with logarithmic increments (6, 8, 10, 15, 26, 60 and 100). Von Frey hairs were applied perpendicularly to the plantar surface with sufficient force to cause slight buckling against the paw, and held for approximately 2–4 sec. Stimuli were presented at intervals of several seconds, allowing for apparent resolution of any behavioral responses to previous stimuli. To evaluate neutrophil infiltration, mieloperoxidase activity (MPO) was evaluated in paw skin sample, as previously described [31]. Briefly, sample was homogenized in acetate buffer (80 mM, pH 5.5) containing 0.5 % HTAB and centrifuged at 16.000 xg during 20 min at 4 °C. After, 10 μL of supernatant were added to 200 μL of acetate buffer and 20 μL of TMB (18.4 mM) and incubated at 37 °C for 3 min. To stop the reaction, the microplates were taken to the ice bath and 30 μL of acetic acid were added. The color formed was assessed at 630 nm and the results were expressed as optical density per mg of tissue (OD/mg tissue).

### Isolation of lymphocytes from blood

Rats were anesthetized with isoflurane and blood was collected by cardiac puncture. Blood was collected with 7.2 mg dipotassium EDTA as anticoagulant and lymphocyte-rich mononuclear cell were isolated from blood collected with Ethylenediamine tetra acetic acid (EDTA) and separated on Ficoll-Histopaque density [32]. The percentage of lymphocytes was superior to 93 %, as previously described [33]. The integrity of lymphocytes preparation was confirmed by determining the lactate dehydrogenase (LDH) activity in intact and disrupted lymphocytes using the kinetic method of the Labquest apparatus (Diagnostics Gold Analyzer). The procedure was repeated before and after the incubation period. The protocol was carried out according to the manufacturer’s instructions. Triton X-100 (1 %, final concentration) was used to disrupt the lymphocytes preparation. The enzymatic activity is expressed as units per liter, and one unit (1U) corresponds to 1 μmol of NADH formed per minute per liter. The resultant lymphocytes samples were used immediately for enzymatic assays.

### Protein determination

Protein was measured by the Comassie Blue method according to Bradford [34] using serum albumin as standard.

### E-NTPDase activity determination

E-NTPDase activity in lymphocytes was determined as previously described by Leal et al. [35], in which the reaction medium contained 0.5 mM CaCl_2_, 120 mM NaCl, 5 mM KCl, 60 mM glucose and 50 mM Tris–HCl buffer at pH 8.0, with a final volume of 200 μL. Twenty microliters of the intact mononuclear cells suspended in saline solution was added to the reaction medium (2–4 μg of protein), and pre-incubated for 10 min at 37 °C; incubation proceeded for 70 min. The reaction was initiated by the addition of substrate (ATP or ADP) at a final concentration of 2.0 mM and stopped with 200 μL of 10 % trichloracetic acid (TCA). The released inorganic phosphate (Pi) was assayed by a method previously described by Chan et al. [36] using malachite green as colorimetric reagent and KH_2_PO_4_ as standard. Controls were carried out by adding the enzyme preparation after TCA addition to correct for non-enzymatic nucleotide hydrolysis. All samples were run in triplicate and the specific activity is reported as nmol of Pi released/min/mg of protein.

### Adenosine deaminase activity determination (ADA)

ADA activity in lymphocytes was measured by the method of Giusti and Galanti [37], which is based on the direct measurement of ammonia produced when ADA acts in excess of adenosine. Briefly, 25 μL of lymphocytes reacted with 21 mM of the substrate (adenosine), pH 6.5, and incubation was carried out for 1 h at 37 °C. The reaction was stopped by adding 106 mM and 167.8 mM sodium nitroprussiate and hypochlorite solution. Ammonium sulfate 75 μM was used as ammonium standard. All the experiments were performed in triplicate and the values were expressed in U/L for ADA activity. One unit (1U) of ADA is defined as the amount of enzyme required to release 1 mmol of ammonia per minute from adenosine at standard assay conditions.

### Separation of blood serum

Rats were anesthetized with isoflurane and blood was collected by cardiac puncture. The blood samples were collected in tubes without anticoagulant and after the clot formation were centrifuged at 1400 g for 15 min at room temperature. The resultant serum samples were aliquoted in microtubes and kept on ice until the purines quantification.

### Purine level measurement

The quantitative determination of adenine nucleotides and adenosine levels were performed in serum by HPLC. At first, proteins were denatured by the addition of 0.6 mol/L of percloric acid. Then, all samples were centrifuged (14,000 × g for 10 min). The obtained supernatants were neutralized with 4 N KOH and clarified with a second centrifugation (14,000 × g for 15 min). Aliquots of 40 μL were applied to a reversed-phase HPLC system using a 25 cm C18 Shimadzu column (Shimadzu, Japan) at 260 nm with a mobile phase containing 60 mM KH_2_PO_4_, 5 mM tetrabutylammonium chloride, pH 6.0, in 30 % methanol according to a method previously described by Voelter [38]. The peaks of purines (ATP, ADP, AMP and adenosine) were identified by their retention times and quantified by comparison with standards. Results are expressed as nmoles of the different compounds per mL of serum.

### Statistical analysis

Data were analyzed by a one- or two-way ANOVA. Post hoc analyses were carried out by the Student-Newman-Keuls (SNK) Multiple Comparison Test. Purine level were log-transformed to achieve normal distribution of data. A probability of *P <* 0.05 was considered significant. All data are expressed as mean ± Standard Error of the Mean (SEM).

## Results

### Evidences of arthritis induction and effect of *U. tomentosa* extract

**Fig. 1:**
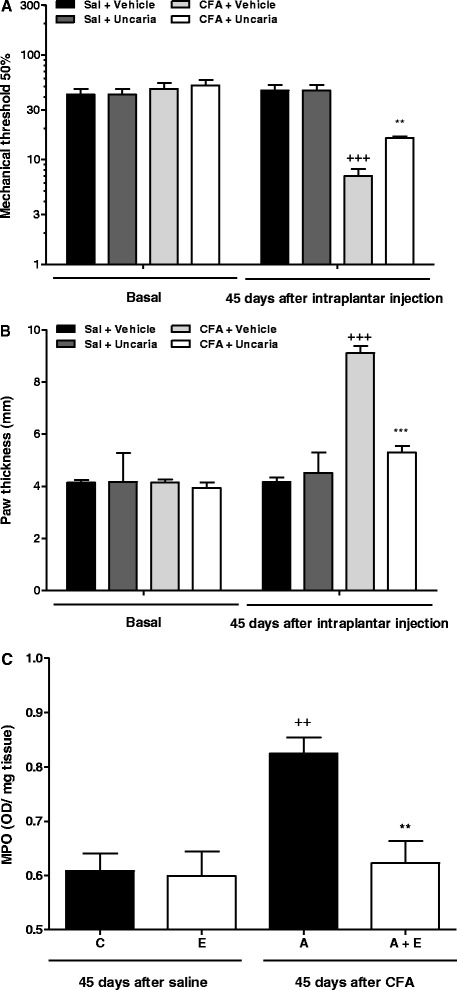
Evidence of arthritis induction and the effect of *U. tomentosa* treatment (150 mg/kg, 2 times/day, v.o.) for 45 days on the inflammatory process induced by CFA. Mechanical sensitivity (**a**) and paw edema (**b**) before (Basal) and after intraplantar injection (saline or CFA), as well as (**c**) MPO activity. “+” and “*” represent statistical difference in relation to control and arthritic group, respectively (*P* < 0.05). Bars represent means ± SEM. One Way ANOVA followed by Student Newman Keuls (SNK)

As shown in Fig. 1, CFA injection was able to increase mechanical sensitivity (mechanical allodynia) (Fig. 1a), paw thickness (Fig. 1b) and MPO activity (Fig. 1c) in 72.4 %, 120.1 % and 35.3 %, respectively, when compared to baseline, characterizing an arthritic process. After 45 days of treatment, *U. tomentosa* extract was able to partially reverse the mechanical allodynia (23.1 ± 2.7 %, *P <* 0.01), edema (21.6 ± 5.7 %, *P <* 0.001) and MPO activity (35.5 ± 5.2 %, *P* < 0.01).

### HPLC analysis of *U. tomentosa* extract

**Fig. 2:**
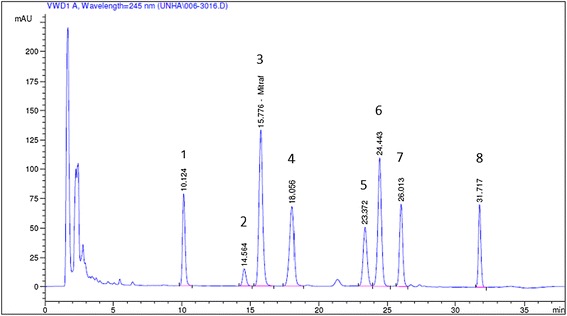
HPLC fingerprint analysis of root dry extract from *U. tomentosa*. Uncarine D (1), uncarine F (2), mytraphyilline (3), rhynchophylline (4), isomytraphylline (5), uncarine C (6), isorhyncophylline (7) and uncarine E (8)

The HPLC analysis of the *U. tomentosa* root dry extract used in the present investigation is presented in Fig. 2. The extract has a content of 0.49 % of pentaciclic and tetraciclic oxindole alkaloids. The concentrations of each one were as follows: uncarine D – 0.05 %, uncarine F – 0.01 %, mytraphyilline – 0.12 %, rhynchophylline – 0.07 %, isomytraphylline– 0.05 %, uncarine C – 0.10 %, isorhyncophylline – 0.05 % and uncarine E – 0.04 %.

### E-NTPDase activity determination

**Fig. 3:**
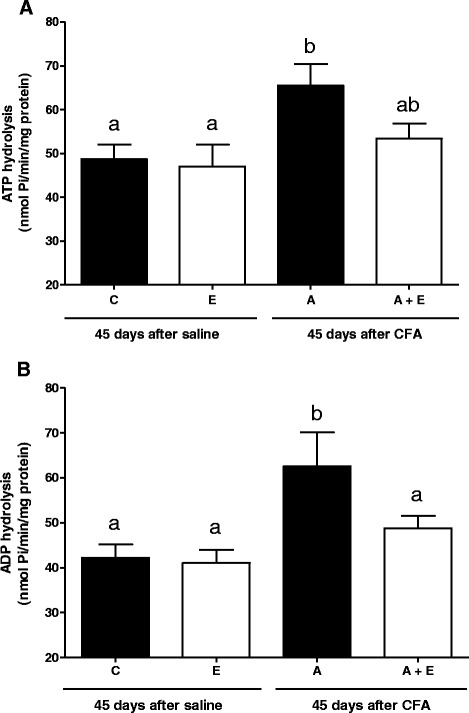
ATP (**a**) and ADP (**b**) hydrolysis in lymphocytes of Complete Freund’s Adjuvant (CFA)- induced arthritis rats and treated for 45 days with *Uncaria tomentosa* extract in the dose of 150 mg/kg, 2 times/day. Enzyme specific activities are reported as nmol of Pi released/min/mg of protein. Groups: C (control), E (extract), A (arthritis) and A + E (arthritis + extract). Bars represent mean ± S.E.M. (^a,b^) Indicates a significant *P* < 0.05, with n = 7 (one-way ANOVA-Newman-Keuls Multiple Comparison Test)

Figure 3 shows the effect of oral administration of *U. tomentosa* extract on ATP and ADP hydrolysis by E-NTPDase in lymphocytes of rats submitted to an experimental adjuvant arthritis model. Results of lymphocytes E-NTPDase activity with ATP as substrate are shown in Fig. 3a. The hydrolysis of ATP was altered in rats with arthritis (A) (65.5 nmol of Pi/min/mg of protein; SEM = 4.8; *n* = 7; *P <* 0.05), demonstrating that ATP hydrolysis was increased by 25.7 % when compared to the control group (C) (48.7 nmol of Pi/min/mg of protein; SEM = 3.2; *n* = 7; *P <* 0.05) and by 28.2 % when compared to the extract group (E) (47 nmol of Pi/min/mg of protein; SEM = 4.9; *n* = 7; *P <* 0.05). However, two-way ANOVA showed no significant interaction [F(1,15) = 1.458; *p* = 0.246; *n* = 7] among the variables. In addition, results obtained for the lymphocytes E-NTPDase activity with ADP as substrate are shown in Fig. 3b, where the ADP hydrolysis was also increased by 32.5 % in the A group (62.4 nmol Pi/min/mg; SEM = 7.6; *n* = 7; *P <* 0.05) when compared to C (42.1 nmol of Pi/min/mg of protein; SEM = 3.0; *n* = 7; *P <* 0.05), in 34.3 % when compared to E group (41.0 nmol of Pi/min/mg of protein; SEM = 2.9; *n* = 7; *P <* 0.05) and in 22 % when compared to A + E group (48.7 nmol of Pi/min/mg of protein; SEM = 2.7; *n* = 7; *P <* 0.05). Two-way ANOVA showed no significant interaction [F(1,13) = 2.606; *p* = 0.130; *n* = 7]. The results of lymphocytes E-NTPDase activity in the group E with both ATP (47.0 nmol of Pi/min/mg of protein; SEM = 4.9; *n* = 7; *P <* 0.05) and ADP (41.0 nmol of Pi/min/mg of protein; SEM = 2.9; *n* = 7; *P <* 0.05) as substrate were similar to ATP (48.7 nmol of Pi/min/mg of protein; SEM = 3.2; *n* = 7; *P <* 0.05) and ADP hydrolysis in control group (42.1 nmol of Pi/min/mg of protein; SEM = 3.0; *n* = 7; *P <* 0.05), showing that in healthy rats the extract did not alter the E-NTPDase activity.

### Adenosine deaminase activity determination (ADA)

**Fig. 4:**
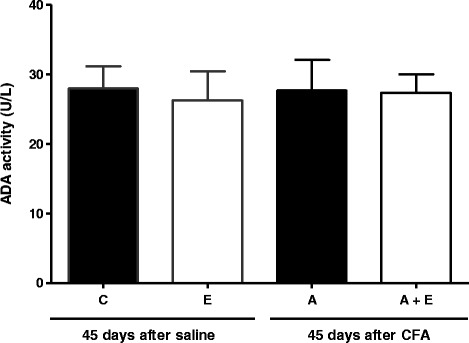
Adenosine hydrolysis in lymphocytes of Complete Freund’s Adjuvant (CFA)-induced arthritis rats and treated for 45 days with *Uncaria tomentosa* extract in the dose of 150 mg/kg, 2 times/day. Enzyme activities are reported as U/L. Groups: C (control), E (extract), A (arthritis) and A + E (arthritis + extract). Bars represent mean ± S.E.M. (^a,b^) Indicates a significant *P* < 0.05, with n = 7 (one-way ANOVA-Newman-Keuls Multiple Comparison Test)

Results obtained for adenosine hydrolysis by E-ADA are shown in Fig. 4. The adenosine hydrolysis was not altered. The groups showed no significant alterations in the E-ADA activity when adenosine was used as substrate. Two-way ANOVA showed no significant interaction [F(1,15) = 1.572, *p* = 0.229, *n* = 7].

### Purine level measurement

Purine levels in serum were measured by HPLC and were showed in Table 1. Levels of ATP, ADP, AMP and adenosine showed no significant alterations in the A group when compared to control. The levels of these nucleotides and nucleoside were also normal in the E group when compared to control, showing that the extract did not interfere in the purine level. However, in the A + E group, the level of ATP was decreased by 14.4 % when compared to C and A groups and in 12.7 % comparing to E group, whereas ADP levels were increase by 7.2 %, 4.6 % and 7.2 % regarding to C, E, and A groups, respectively. The adenosine levels in in the A + E group were also decreased by 17.8 % in comparison to control.

**Table 1:** Purine levels in arthritic rats serum and treated for 45 days with *Uncaria tomentosa* extract

	C	E	A	A + E
	(log of nmol/ml)	(log of nmol/ml)	(log of nmol/ml)	(log of nmol/ml)
ATP	1.04 ± 0.003^a^	1.02 ± 0.01^a^	1.04 ± 0.02^a^	0.89 ± 0.01^b^
ADP	1.25 ± 0.002^a^	1.28 ± 0.01^a^	1.25 ± 0.01^a^	1.34 ± 0.001^b^
AMP	1.00 ± 0.005^a^	1.05 ± 0.008^a^	1.04 ± 0.008^a^	1.02 ± 0.02^a^
Adenosine	1.35 ± 0.001^a^	1.29 ± 0.02^ab^	1.33 ± 0.01^ab^	1.11 ± 0.08^b^

Adenine nucleotides and adenosine levels measurement in serum of Complete Freund’s Adjuvant (CFA)-induced arthritis rats and treated for 45 days with *Uncaria tomentosa* extract (150 mg/kg; 2 times/day). Purine levels measurement were log-transformed and are reported as log of nmol/ml. Groups: C (control), E (extract), A (arthritis) and A + E (arthritis + extract). Bars represent mean ± S.E.M

(^a,b^) Indicates difference among the groups, *P* < 0.05 with n = 7 (two-way ANOVA-Newman-Keuls Multiple Comparison Test)

## Discussion

Experimental animal models of chronic diseases allow a better understanding of the physiopathological processes and also the evaluation of potential new therapies. Based on that, the model of arthritis induced by CFA in rats is widely used in the research of new therapies for chronic inflammatory arthropathies, such as rheumatoid arthritis [39]. Cat’s claw (*Uncaria tomentosa*) has been widely used for the treatment of arthritis, rheumatism and other inflammatory diseases due to its well known anti-inflammatory effects [39–41]. This plant contains a series of secondary metabolites, such as oxindole alkaloids, polyphenols (flavonoids, proanthocyanidins, tannins), quinovic acid glycosides, polyhydroxylated triterpenes and saponins, but its anti-inflammatory activity is related to more than one metabolite acting in synergy [4, 5, 42, 43]. Here, the HPLC analysis revealed that mitraphylline was the preponderant alkaloid present in the *U. tomentosa* extract used for this research. In fact, Rojas-Duran et al. [3] reported that the major alkaloid of *U. tomentosa* was mytraphylline and evaluated its biological relevance demonstrating that it was able to impair the liberation of the interleukins 1α, 1β, 4, 17 and TNF-α, key molecules involved in inflammatory responses. Since the inflammatory action is closely associated to the immune process [44], benefits of this plant to the immune system, such as in purinergic signaling present in lymphocytes, could also be expressed as an anti-inflammatory action. However, a direct relationship between the components of this plant and purine nucleotides has not yet been reported in the literature.

In the present study, we induced arthritis in animals by the use of CFA and the inflammatory process was confirmed through the measurement of increased paw thickness, mechanical thresholds and MPO activity (neutrophil marker), which characterizes an arthritic process. After that, we analyzed if *U. tomentosa* extract would be able to reverse this process, and it showed to be partially effective. Similar results were also observed with carrageenan-induced paw edema demonstrating that both hydro-alcoholic and aqueous extract have anti-inflammatory activity by decreasing the carrageenan-induced increase in paw volume when compared with control rats [44–46]. Human tests with *U. tomentosa* were carried out in patients with osteoarthritis and rheumatoid arthritis and demonstrated that it was able to reduce pain, morning stiffness and swelling joints [47–49].

Extensive tissue damage in inflammatory processes may lead to a significant increase in the levels of purine and pyrimidine nucleotides within the involved sites, probably contributing to the amplification of the inflammatory reaction [50]. Extracellular ATP can act as a damage-associated molecular pattern, given that it is normally confined to intracellular sites but can be released at high levels following cell lysis, infection, or via regulated efflux. ATP released into the extracellular space can modulate the immune response through its capacity to bind and activate multiple nucleotide receptor family members [51, 52]. In addition, the purinergic system is also composed by a complex of ectoenzymes including E-NTPDase and E-ADA that are responsible for extracellular nucleotide hydrolysis.

The results of the present study showed an increased E-NTPDase activity in rats with arthritis when compared to control animals corroborating with Becker et al. [53] which found increased E-NTPDase activity in platelets of patients with arthritis. According to a study previously published by our research group, the E-NTPDase activity (both ATP and ADP substrate) in lymphocytes was also altered in patients with RA. The ATP hydrolysis presented a two-fold increase, while ADP hydrolysis was 80 % increased when compared to the control group. [54]. Moreover, many other studies have shown that the E-NTPDase and E-ADA have significant roles in immune response. Alterations in their activities have been observed in some autoimmune diseases such as multiple sclerosis, lupus and diabetes [55–57]. Increased activity of E-NTPDase leads to increased ATP and ADP hydrolysis and, as a compensatory mechanism, leads to the maintenance of their appropriate levels, since high concentrations of ATP in the extracellular medium activate the pro-inflammatory purinergic P2X7 receptors and contributes to tissue damage and inflammation [58].

Since these enzymes act in a cascade, we can suggest that the 5′-nucleotidase activity in arthritic rats could be also increased, resulting in a greater amount of adenosine in the extracellular medium to compensate the pro-inflammatory effects of ATP. However, in these arthritic animals, the levels of ATP, ADP, AMP and adenosine are normal in the extracellular medium as well as E-ADA activity. We can assume that adenosine is being produced in excess by the supposed increased activity of 5′-nucleotidase. This adenosine could be binding to specific receptors expressed on the cell surface exercising its anti-inflammatory function and maintaining the levels of adenosine normal on extracellular medium. Thus, adenosine acts as a negative feedback signal to counteract ATP-mediated immune stimulation, preventing uncontrolled inflammation and decreasing the collateral damage to healthy tissues [59]. The anti-inflammatory properties of *U. tomentosa* have been described, hence we evaluated the effect of the *U. tomentosa* dry extract on the metabolism of adenine nucleotides. In healthy animals treated with the extract, the activities of E-NTPDase and E-ADA remained at basal levels, what was also confirmed by serum purine levels, which were in normal concentration in the extracellular medium.

In arthritic rats treated with *U. tomentosa* dry extract, the increase on the E-NTPDase activity was prevented, while the purine levels on serum showed decreased ATP levels and increased ADP levels. It seems that low-level purinergic signaling induced by nucleotides at decreased concentrations, modulates ongoing inflammatory and immune responses by P2 receptors [60]. At low concentration, extracellular ATP possesses affinity for P2Y receptor subtype on the surfaces of lymphocytes. These purinergic receptors, when stimulated, develop a down-modulation of pro-inflammatory cytokines and stimulate the Th2 immune response, leading to the production of anti-inflammatory cytokines, protection from oxidative damage and down-production of oxygen radicals in whole blood [16]. P2Y receptor signaling may therefore be an important stop signal to prevent excessive stimulation of inflammation and avoid conditions that might favor autoimmunity [61].

Among the various classes of chemical constituents found in *U. tomentosa* plant, oxindole alkaloids are regarded as the most important [43]. The alkaloid profile in *U. tomentosa* is important because of its pharmacological properties and because the pentacyclic oxindole alkaloids are directly associated with immunostimulatory properties [62]. The anti-inflammatory activity of traditional extracts made from *U. tomentosa* is well documented [63–65]. Mitraphylline is the most ubiquitous alkaloid being present in *Uncaria* species [62], being also the major compound present in our lot of *U. tomentosa* extract. Mitraphylline is considered as a new lead compound for the development of anti-inflammatory treatment, being the most chemical effective component and responsible for suppression of inflammation parameters. In one previous study, this compound inhibited around 50 % of the release of pro-inflammatory interleukins, and its activity was similar to dexamethasone [3]. Therefore, these metabolites are certainly contributing to decrease the inflammatory process and regulate the purinergic signaling in this data.

Therefore, in this model, these metabolites probably contribute to decrease the inflammatory process and regulate the purinergic signaling. No changes were observed in the ADA activity, but a decrease in adenosine levels was observed in serum of the arthritic rats that received *U. tomentosa* extract. Corroborating our results, a study previously published by our research group [52] has showed that the level of adenosine in serum of RA patients was also decreased. In addition, ADP levels showed to be increased in this same group. It is possible that the adenylate kinase (EC 2.7.4.3) could be activated in an attempt to reconstitute the pool of ADP. As proposed by Yegutkin et al. [66], an opposite pathway could lead to the recovery of adenine nucleotides, since adenylate kinase was identified as another key player in the metabolism of circulating ADP. This extracellular ADP could be linking itself to P2Y receptor, the adenine-nucleotide-preferring receptors mainly responding to ADP, and leading the anti-inflammatory response.

## Conclusion

In summary, our data demonstrate that the *U. tomentosa* extract was able to reduce partially the mechanical thresholds, paw thickness and MPO activity in a model of induced arthritis. In addition, the extract was able to prevent the increase on the E-NTPDase activity in lymphocytes of rats submitted to an experimental adjuvant arthritis model. In view of this, *U. tomentosa* extract had an actual effect against arthritis and for the first time we demonstrate that the purinergic signaling is involved in these responses.

## Abbreviations

ADP: Adenosine diphosphate
AMP: Adenosine monophosphate
ATP: Adenosine triphosphate
CFA: Complete Freund’s Adjuvant
MPO: Myeloperoxidase

## References

[1] Reinhard KH (1999). *Uncaria tomentosa* (Willd.) D.C.: Cat’s claw, uña de gato, or saventaro. J Altern Complement Med.

[2] Patidar A, Birla D, Patel V, Chaturvedi M, Manocha N (2014). A review of on advantages of natural analgesics over conventional synthetic analgesics. Int J Pharm Life Sci.

[3] Rojas-Durana R, Gonza’lez-Aspajoa G, Ruiz-Martel C, Bourdy G, Doroteo-Ortega VH, Alban-Castillo J, Robert G, Auberger P, Deharo E (2012). Anti-inflammatory activity of Mitraphylline isolated from Uncaria tomentosa bark. J Ethnopharmacol.

[4] Wagner H, Kreutzkamp B, Jurcic K (1985). Die Alkaloide von *Uncaria tomentosa* ihre phagozytosesteigernde wirkung. Planta Med.

[5] Aquino R, De Simone F, Pizza C, Conti C, Stein ML (1989). Plant Metabolites structure and in vitro antiviral activity of quinovic acid glycosides from *Uncaria tomentosa* and *Guettarda platypoda*. J Nat Prod.

[6] Aquino R, Vicenzo F, Francesco S (1991). Plant Metabolites. New compounds and anti inflamatory activity of *Uncaria tomentosa*. J Nat Prod.

[7] Yoshimoto K, Sato H, Ohtake M, Laus G, Brössner D, Keplinger K (1997). Alkaloids of Peruvian *Uncaria tomentosa*. Phytochemistry.

[8] Falkiewicz B, Łukasiak J (2001). Vilcacora [*Uncaria tomentosa* (Willd.) DC. and *Uncaria guianensis* (Aublet) Gmell.]—a review of published scientific literature. Case Rep Clin Pract Rev.

[9] Choy EH, Panayi GS (2001). Cytokine pathways and joint inflammation in rheumatoid arthritis. N Engl J Med.

[10] MacGregor AJ, Silman AJ, Hochberg MC (2004). Rheumatoid arthritis and other synovial disorders: Classification and epidemiology. Rheumatology.

[11] Bush KA, Kirkham BW, Walker JS (2002). The in vivo effects of tumor necrosis factor blockade on the early cell mediated immune events and syndrome expression in rat adjuvant arthritis. Clin Exp Immunol.

[12] Hegen M, Keith JC, Collins M, Nickerson-Nutter CL (2008). Utility of animal models for identification of potential therapeutics for rheumatoid arthritis. Ann Rheum Dis.

[13] Iain BM, Georg S (2007). Cytokines in the pathogenesis of rheumatoid arthritis. Nat Rev Immunol.

[14] Luttikhuizen DT, Harmsen MC, de Leij LFMH, van Luyn MJA (2004). Expression of P2 receptors at site of chronic inflammation. Cell Tissue Res.

[15] Ralevic V, Burnstock G (2003). Involvement of purinergic signaling in cardiovascular diseases. Drug News Perspect.

[16] Bours M, Swennen E, Di Virgilio F, Cronstein BN, Dagnelie PC (2006). Adenosine 5′ triphosphate and adenosine as endogenous signaling molecules in immunity and inflammation. Pharmacol Ther.

[17] Langston H, Ke Y, Gewirtz A, Dombrowski K, Kapp J (2003). Secretion of IL-2 and IFN-y, but not IL-4, by antigen-speci.c T cells requires extracellular ATP. J Immunol.

[18] Robson SC, Sévigny J, Zimmermann H (2006). The E-NTPDase family of ectonucleotidases: structure function relationships and pathophysiological significance. Purinergic Signal.

[19] Zimmermann H, Mishra S, Shukla V, Langer D, Gampe K, Grimm I, Delic J, Braun N (2007). Ecto-nucleotidases, molecular properties and functional impact. An R Acad Nac Farm.

[20] Yegutkin GG (2008). Nucleotide and nucleoside converting ectoenzymes: important modulators of purinergic signalling cascade. Biochim Biophs Acta.

[21] Pulte E, Broekman M, Olson K, Drosopoulos JHF, Kizer JR, Islam N, Marcus AJ (2007). CD39/NTPDase-1 activity and expression in normal leucocytes. Thromb Res.

[22] Barankiewicz J, Dosch HM, Cohen A (1988). Extracellular nucleotide catabolism in human B and T lymphocytes. The source of adenosine production. J Biol Chem.

[23] Franco R, Casadó V, Ciruela F, Saura C, Canela EI, Lluis C (1997). Cell surface adenosine deaminase: much more than an ectoenzyme. Prog Neurobiol.

[24] Bota A, Javier Gella F, Profilis C, Férard G, Hadjivassiliou AG, Hørder M, Schiele F, Segura R, Canalias F (2001). Production and certification of an enzyme reference material for adenosine deaminase 1 (BCR 647). Clin Chim Acta.

[25] Antonioli L, Fornai M, Colucci R, Ghisu N, Tuccori M, Del Tacca M, Blandizzi C (2008). Pharmacological modulation of adenosine system: novel options for treatment of inflammatory bowel diseases. Inflamm Bowel Dis.

[26] SBCAL (2009). Sociedade Brasileira de Ciências em Animais de Laboratório.

[27] Sauzem PD, Sant’Anna GS, Machado P, Duarte MM, Ferreira J, Mello CF, Beck P, Bonacorso HG, Zanatta N, Martins MA, Rubin MA (2009). Effect of 5-trifluoromethyl-4,5-dihydro-1H-pyrazoles on chronic inflammatory pain model in rats. Eur J Pharmacol.

[28] Bertol G, Franco L, Oliveira BH (2012). HPLC Analysis of Oxindole Alkaloids in *Uncaria tomentosa*: Sample Preparation and Analysis Optimisation by Factorial Design. Phytochem Anal.

[29] Cao YQ, Mantyh PW, Carlson EJ, Gillespie A, Epstein CJ, Basbaum A (1998). Primary afferent tachykinins are required to experience moderate to intense pain. Nature.

[30] Dixon WJ (1980). Efficient analysis of experimental observations. Ann Rev Pharmacol Toxicol.

[31] Suzuki K, Ota H, Sasagawa S, Sakatani T, Fujikura T (1983). Assay method for myeloperoxidase in human polymorphonuclear leukocytes. Anal Biochem.

[32] Böyum A (1968). Isolation of mononuclear cells and granulocytes from human blood. Isolation of monuclear cells by one centrifugation, and of granulocytes by combining centrifugation and sedimentation at 1 g. Scand J Clin Lab Invest Suppl.

[33] Jaques JA, Peres Rezer JF, Ruchel JB, Gutierres J, Bairros AV, Gomes Farias IL, da Luz SC A, Mello Bertoncheli C, Chitolina Schetinger MR, Morsch VM, Leal DB (2011). A method for isolation of rat lymphocyte-rich mononuclear cells from lung tissue useful for determination of nucleoside triphosphate diphosphohydrolase activity. Anal Biochem.

[34] Bradford MM (1976). A rapid and sensitive method for the quantitation of microgram quantities of protein utilizing the principle of protein-dye binding. Anal Biochem.

[35] Leal DBR, Streher CA, Neu TN, Bitencourt FP, Leal CAM, Silva JEP, Morsch VM, Schetinger MRC (2005). Characterization of NTPDase (NTPDase1; ecto-apyrase; ecto-diphosphohydrolase; CD39; E.C. 3.6.1.5) activity in humans lymphocytes. Biochim Biophys Acta.

[36] Chan KM, Delfert D, Junger KD (1986). A direct colorimetric assay for Ca2+ stimulated ATPase activity. Anal Biochem.

[37] Giusti G, Galanti B, Bergmeyer HU (1984). Colorimetric Method. Methods of enzymatic analysis.

[38] Voelter W, Zech K, Arnold P, Ludwig G (1980). Determination of selected pyrimidines, purines and their metabolites in serum and urine by reverse-phase ion-pair chromatography. J Chromatogr.

[39] Akesson C, Lindgren H, Pero RW, Leanderson T, Ivars F (2005). Quinic acid is a biologically active component of the *Uncaria tomentosa* extract C-Med 100. Int Immunopharmacol.

[40] Jurgensen S, Dalbó S, Angers P, Santos AR, Ribeiro do Valle RM (2005). Involvement of 5-HT2 receptors in the antinociceptive effect of *Uncaria tomentosa*. Pharmacol Biochem Behav.

[41] Pilarski R, Zieliński H, Ciesiołka D, Gulewicz K (2006). Antioxidant activity of ethanolic and aqueous extracts of *Uncaria tomentosa* (Willd.) DC. J Ethnopharmacol.

[42] Aquino R, De Simone F, Pizza C, Vincieri F, Gacs BE (1990). New polyhydroxylated triterpenes from *Uncaria tomentosa*. J Nat Prod.

[43] Laus G, Brossner D, Keplinger K (1997). Alkaloids of peruvian *Uncaria tomentosa*. Phytochemistry.

[44] Aguilar JL, Rojas P, Marcelo A, Plaza A, Bauer R, Reininger E, Klaas CA, Merfort I (2002). Anti-inflamatory activity of two extracts of *Uncaria tomentosa* (Rubiaceae). J Ethnopharmacol.

[45] Joe B, Griffiths MM, Remmers EF, Wilder RL (1999). Animal models of rheumatoid arthritis and related inflammation. Cur Rheumat R.

[46] Keplinger K, Laus G, Wurm M, Dierich MP, Teppner H (1999). *Uncaria tomentosa* (Wild). Ethnomedicinal Uses and new pharmacological, toxicological and botanical results. J Ethnopharmacol.

[47] Castañeda O, León G, León D, Calvoh A, Chávez J, Escalante J, Luza A, Quevedo H, Sedano O, Vega E (1998). Uña de Gato en Artritis Reumatóide: Estúdio doble cego, en comparación com placebo. Rev Peru Reumatol.

[48] Piscoya J, Rodriguez Z, Bustamante SA, Okuhama NN, Miller MJ, Sandoval M (2001). Efficacy and safety of freeze-dried cat’s claw in osteoarthritis knee: mechanism of actions of the species *Uncaria guianensis*. Inflamm Res.

[49] Mur E, Hartig F, Eibl G, Schirmer M (2002). Randized double blind trial of an extract from the pentacyclic alkaloid-chemotype of *Uncaria tomentosa* for the treatment of rheumatoid arthritis. J Rheumatol.

[50] Miyara M, Sakaguchi S (2007). Natural regulatory T cells: mechanisms of suppression. Trends Mol Med.

[51] la Sala A, Ferrari D, Di Virgilio F, Idzko M, Norgauer J, Girolomoni G (2003). Alerting and tuning the immune response by extracellular nucleotides. J Leukoc Biol.

[52] Gordon JL (1986). Extracellular ATP: effects, sources and fate. Biochem J.

[53] Becker LV, Rosa CS, Souza VCG, Bagatini MD, Casali EA, Leal CAM, Silva JCN, Moretto MB, Pinheiro FV, Morsch VM, Schetinger MRC, Leal DBR (2010). Activities of enzymes that hydrolyze adenine nucleotides in platelets from patients with rheumatoid arthritis. Clin Biochem.

[54] Jaques JAS, Becker LV, Souza VCG, Leal CAM, Bertoldo TMD, Pinheiro KV, Morsch VM, Schetinger MRC, Leal DBR (2013). Activities of enzymes that hydrolyze adenine nucleotides in lymphocytes from patients with rheumatoid arthritis. Cell Biochem Funct.

[55] Loza MJ, Anderson AS, O’Rourke KS, Wood J, Khan IU (2011). T-cell specific defect in expression of the NTPDase CD39 as a biomarker for lupus. Cell Immunol.

[56] Schmatz R, Schetinger MR, Spanevello RM, Mazzanti CM, Stefanello N, Maldonado PA, Gutierres J, Correa Mde C, Girotto E, Moretto MB, Morsch VM (2009). Effects of resveratrol on nucleotide degrading enzymes in streptozotocin-induced diabetic rats. Life Sci.

[57] Spanevello RM, Mazzanti CM, Schmatz R, Thome G, Bagatini M, Correa M, Rosa C, Stefanello N, Belle LP, Moretto MB, Oliveira L, Morsch VM, Schetinger MR (2010). The activity and expression of NTPDase is altered in lymphocytes of multiple sclerosis patients. Clin Chim Acta.

[58] Di Virgilio F (1995). The P2Z purinoreceptor: intriguing role in immunity, inflammation and cell death. Immunol Today.

[59] Gessi S, Varani K, Merighi S, Fogli E, Sacchetto V, Benini A, Leung E, Mac-Lennan S, Borea PA (2007). Adenosine and lymphocyte regulation. Purinergic Signal.

[60] Di Virgilio F, Ferrari D, Idzko M, Panther E, Norgauer J, La Sala A, Extracellular GG, ATP (2003). P2 receptors, and inflammation. Drug Devel R.

[61] Di Virgilio F, Boeynaems JM, Robson SC (2009). Extracellular nucleotides as negative modulators of immunity. Cur Op Pharmacol.

[62] Heitzman ME, Neto CC, Winiarz E, Vaisberg AJ, Hammond GB (2005). Ethnobotany, phytochemistry and pharmacology of *Uncaria* (Rubiaceae). Phytochemistry.

[63] Erowele GI, Kalejaiye AO (2009). Pharmacology and therapeutic uses of cat’s claw. Am J Health Syst Pharm.

[64] Urdanibia I, Estrada O, Ibarra C, Michelangeli F, Milano B, Taylor P (2013). Anti-inflammatory and anti-tumour effects of two species of Cat’s claw (Uncaria). Planta Med.

[65] Urdanibia I, Estrada O, Ibarra C, Michelangeli F, Ruiz MC, Taylor P (2012). Anti-inflammatory effects of different preparations of cat’s claw. Planta Med.

[66] Yegutkin GG, Henttinen T, Samburski SS, Spychala J, Jalkanen S (2002). The evidence for two opposite, ATP-generating and ATP-consuming, extracellular pathways on endothelial and lymphoid cells. Biochem J.

